# Immunomodulatory Effects of *Ganoderma lucidum* Bioactive Compounds on Gut–Brain and Gut–Liver Axis Disorders

**DOI:** 10.3390/cimb47100842

**Published:** 2025-10-14

**Authors:** Liting Zhao, Zijun Geng, Ying Wang, Jiawei Wen, Da Liu

**Affiliations:** 1 School of Pharmacy, Changchun University of Chinese Medicine, Changchun 130117, China; zlt15020747022@163.com (L.Z.); jimmyg_98@163.com (Z.G.); 23203560022@stu.ccucm.edu.cn (Y.W.); 2 Institute of Economic Botany, Jilin Academy of Agricultural Sciences, Changchun 130033, China; 3 Public Laboratory Centre, Changchun University of Chinese Medicine, Changchun 130117, China

**Keywords:** *Ganoderma lucidum*, gut–brain axis, gut–liver axis, intestinal flora, immunity

## Abstract

*Ganoderma lucidum* (Lingzhi), a traditional medicinal mushroom, is renowned for its immunomodulatory, anti-inflammatory, and antioxidant properties, primarily attributed to its bioactive components such as polysaccharides and triterpenoids. This review focuses on the mechanisms by which *Ganoderma lucidum* modulates immune responses, particularly in the context of gut–liver–brain axis disorders. Polysaccharides enhance immune function by activating macrophages, natural killer cells, and T cells, thereby promoting phagocytosis and cytokine production. Triterpenoids contribute through anti-inflammatory and antioxidant activities, inhibiting inflammatory mediators and protecting tissues from damage. *Ganoderma lucidum* also influences immune regulation via key signaling pathways, including NF-κB and MAPK, and supports immune tolerance, potentially reducing the risk of autoimmune diseases. Additionally, it modulates gut microbiota, which further impacts systemic immunity. Importantly, polysaccharides and triterpenoids demonstrate promising clinical application prospects in metabolic diseases, inflammatory conditions, neurodegenerative disorders, and cancer immunotherapy, attributed to their multi-target immunomodulatory activities and prebiotic properties. Despite promising applications in treating metabolic, inflammatory, and neurodegenerative diseases, further research is needed to fully elucidate the molecular mechanisms and potential of *Ganoderma lucidum* in precision medicine. This comprehensive analysis underscores the value of *Ganoderma lucidum* as a multifaceted immunomodulatory agent.

## 1. Introduction

*Ganoderma lucidum* is a highly regarded medicinal fungus belonging to the Basidiomycota class, Polyporaceae family, and Ganoderma genus. Currently, there are approximately 120 recognized species of the Ganoderma genus worldwide. *Ganoderma lucidum* is predominantly distributed throughout Asia, characterized by a complex chemical composition, with significant research focused on *Ganoderma lucidum* polysaccharides, triterpenes, and nucleoside compounds [1].

The pharmacological properties of *Ganoderma lucidum* are multifaceted, encompassing the following key aspects [1,2]: 

(1) Antioxidant efficacy: Rich in polysaccharides and triterpenes, *Ganoderma lucidum* acts as a potent free radical scavenger, alleviating oxidative stress and associated tissue damage, which may contribute to delayed aging. 

(2) Anti-inflammatory efficacy: Its triterpenes and polysaccharides significantly suppress inflammatory responses and tissue injury, supporting the management of chronic conditions such as hepatitis, asthma, and allergic rhinitis [3].

(3) Hypoglycemic and hypolipidemic effects: The active constituents of *Ganoderma lucidum* have been shown to promote insulin secretion and enhance insulin sensitivity, thereby resulting in reduced blood glucose levels [4]. They also regulate lipid profiles by lowering cholesterol and triglycerides, aiding in the prevention and management of diabetes and cardiovascular diseases [5,6,7]. 

(4) Immunomodulatory effect: The polysaccharides and triterpenes in *Ganoderma lucidum* exhibit significant immunomodulatory effects by enhancing immune function and increasing the body’s resistance to diseases. Additionally, *Ganoderma lucidum* contains a variety of pharmacologically active components—including triterpenes, polysaccharides, organic germanium, adenine nucleosides, and trace elements—which collectively contribute to strengthening human immunity and overall resistance [8]. *Ganoderma lucidum* has been shown to promote leukocyte proliferation, enhance the levels of immunoglobulins and complement proteins, and activate the activity of natural killer cells and macrophages. 

(5) Antitumor activity: *Ganoderma lucidum* exhibits notable antitumor properties, including the inhibition of tumor cell growth and metastasis. Specifically, *Ganoderma lucidum* polysaccharide peptides have been shown to enhance the ability of dendritic cells to capture, process, and present tumor antigens, which promotes the activation and cytotoxic activity of cytotoxic T cells. Additionally, *Ganoderma lucidum* can improve the efficacy of radiotherapy and chemotherapy while reducing their side effects [9]. 

(6) Antiviral activity: *Ganoderma lucidum* has demonstrated inhibitory effects against novel coronavirus infection. Studies have shown that both *Ganoderma lucidum* water extracts and polysaccharides can significantly suppress SARS-CoV-2 infection in vivo and in vitro. In particular, the *Ganoderma lucidum* polysaccharide RF3 exhibits potent antiviral activity against SARS-CoV-2 in cell culture models, without detectable toxicity to host cells [10]. These findings provide a scientific foundation for the potential application of *Ganoderma lucidum* in the prevention and treatment of infectious diseases.

**Figure 1:**
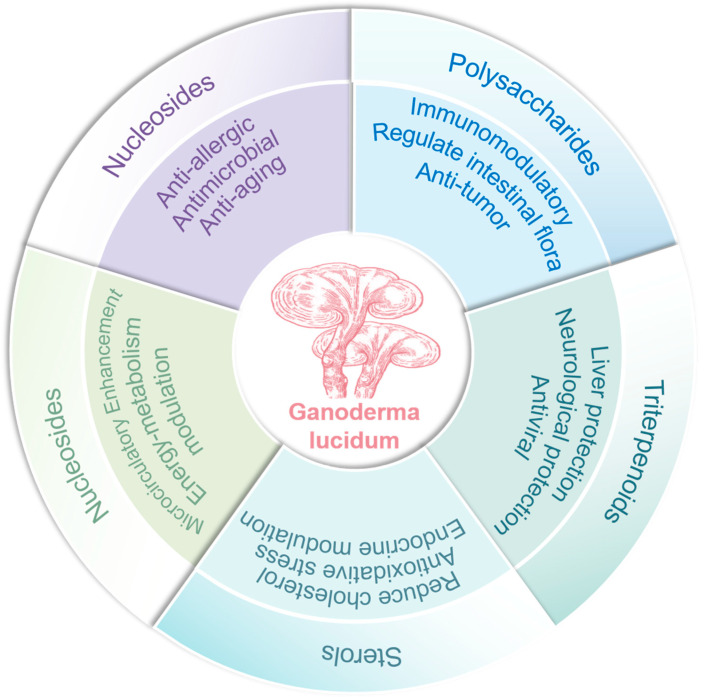
The pharmacological activity of *Ganoderma lucidum*. Core compound classes—polysaccharides, triterpenoids, sterols, and nucleosides—are shown alongside their documented biological functions, such as immunomodulation, antitumor, and hepatoprotective activities, underscoring the synergistic and multi-target nature of its therapeutic effects.

In summary, *Ganoderma lucidum* demonstrates anti-inflammatory, antioxidant, hypoglycemic, hypolipidemic, and immunomodulatory effects (Figure 1). Notably, its diverse mechanisms of immune regulation underlie its broad therapeutic potential, including antitumor activity, prevention of chronic diseases, inhibition of viral infections, and enhancement of immune function. This article primarily focuses on elucidating the crucial roles of *Ganoderma lucidum*’s active constituents in modulating immune responses and metabolic pathways, particularly in diseases associated with the gut–brain axis and gut–liver axis.

## 2. Current Research Status on the Pharmacological Effects and Immunomodulatory Mechanisms of *Ganoderma lucidum*

### 2.1. Chemical Structural Diversity and Bioactivities of Major Bioactive Constituents in Ganoderma lucidum

The medicinal value of *Ganoderma lucidum* primarily arises from its structurally diverse bioactive constituents, among which polysaccharides, triterpenoids, nucleotides, and sterols represent the major classes [1]. Each category possesses distinct chemical characteristics and exhibits considerable structural diversity.

Among these constituents, *Ganoderma lucidum* polysaccharides are macromolecular polymers consisting of monosaccharides, such as glucose, galactose, and mannose, which are primarily interconnected through β-glycosidic bonds. The structural diversity of GLPs is manifested in variations in backbone configuration, branch-chain length and density, as well as higher-order conformations. Notably, the immunomodulatory activity and other bioactivities of GLPs are closely correlated with the presence of β-type glycosidic bonds and the triple-helix conformation [11,12,13]. Similarly, *Ganoderma lucidum* triterpenoids are characteristic bioactive components that display remarkable structural diversity. They are primarily classified into tetracyclic and pentacyclic triterpenoids, as well as those with unique skeletons, such as the ring-opening triterpenoid ganodermanondiol. The tetracyclic triterpenoids possess a lanostane-type tetracyclic framework. Their structural diversity arises from variations in side-chain cyclization, oxidation state, and the number, position, and stereochemistry of functional groups (e.g., hydroxyl, carbonyl, and carboxyl) attached to the core skeleton. These structural intricacies directly underpin the broad spectrum of biological activities exhibited by *Ganoderma lucidum* triterpenoids [14]. The bioactivity of *Ganoderma lucidum* triterpenoids is intimately linked to their precise chemical structures. Studies have demonstrated that the type, quantity, and stereochemistry of oxygen-containing functional groups (such as hydroxyl and carbonyl) at positions C-3, C-7, and C-11 are critical determinants of their pharmacological potency. For example, regarding antitumor activity, Ganoderic Acid A, which features a C-3 hydroxyl group and an 11-oxo structure, has exhibited therapeutic effects against cancer in both in vitro and in vivo studies [15,16]. In terms of anti-inflammatory activity, the presence of a hydroxyl group at the C-3 position is crucial for suppressing the production of inflammatory cytokines [17,18].

In summary, the structural complexities of both polysaccharides and triterpenoids are fundamental to their respective bioactivities. The presence of β-glycosidic bonds and a triple-helix conformation in polysaccharides, as well as the specific functional groups attached to the triterpenoid core, serve as critical determinants of their immunomodulatory and pharmacological activities. These findings underscore the central importance of structure–activity relationships in these two major classes of constituents.

### 2.2. Current Research Status on the Pharmacological Effects of Ganoderma lucidum

*Ganoderma lucidum*, historically regarded as a precious traditional Chinese medicinal herb, occupies a significant position within East Asian traditional medical systems. This fungus contains a diverse array of bioactive components, of which polysaccharides and triterpenoids are recognized as the primary active constituents. Notably, *Ganoderma lucidum* polysaccharides exhibit a wide spectrum of biological activities, including antitumor, immunomodulatory, antioxidant, and anti-inflammatory effects [19]. Studies have demonstrated that *Ganoderma lucidum* polysaccharides exert immunomodulatory effects through multiple mechanisms: (1) Activation of immune cells, including macrophages, T lymphocytes, and B lymphocytes, thereby enhancing both innate and adaptive immune responses. The activation of these cells contributes to increased cytokine production, augmented phagocytic activity, and improved antigen presentation, collectively strengthening the body’s overall immune function [20]. (2) Antioxidant function: They can scavenge free radicals, reducing oxidative stress damage to cells, thereby slowing down the aging process and preventing chronic diseases. (3) Antitumor effect: By modulating the immune system, *Ganoderma lucidum* polysaccharides effectively inhibit tumor cell proliferation and induce apoptosis [21]. *Ganoderma lucidum* triterpenoids exhibit significant antitumor, antiviral, anti-inflammatory, and lipid-lowering effects, effectively inhibiting tumor cell proliferation and inducing apoptosis. Antitumor and anti-inflammatory effects: These compounds have been shown to inhibit tumor cell proliferation and reduce inflammation through inhibition of inflammatory factor production. Cardiovascular protection: By reducing serum lipid concentrations, *Ganoderma lucidum* triterpenoids confer protective effects on the cardiovascular system, reducing the risk of atherosclerosis and hypertension. Antiviral effects: They also demonstrate antiviral efficacy, with studies indicating their ability to inhibit the activity of various viruses [4]. Recent research into the pharmacological effects of *Ganoderma lucidum* has advanced to molecular mechanisms [22,23]. Mechanisms involving the regulation of signaling pathways and gene expression have been elucidated. Specifically, *Ganoderma lucidum* polysaccharides act by modulating immune cell activity through activation of toll-like receptors (TLRs) [24,25]. Moreover, *Ganoderma lucidum* triterpenoids mediate anti-inflammatory and antitumor responses through the inhibition of key signaling pathways, including nuclear factor kappa B (NF-κB) and mitogen-activated protein kinases (MAPK) [26,27]. Collectively, these findings elucidate the multifaceted pharmacological mechanisms of *Ganoderma lucidum* and support its application in clinical therapeutic strategies [28,29].

In conclusion, contemporary research has substantiated the broad-spectrum efficacy of *Ganoderma lucidum*, which is primarily attributed to its polysaccharides and triterpenoids. The multifaceted pharmacological activities of these compounds, ranging from immunomodulation to antitumor effects, are being increasingly elucidated at the molecular level, particularly through their regulation of key signaling pathways. This mechanistic understanding further solidifies the scientific foundation for the potential clinical application of *Ganoderma lucidum*.

### 2.3. Immunomodulation and the Mechanism of Immunomodulatory Effects of Ganoderma lucidum

#### 2.3.1. The Role of Immunomodulation in Disease

Immunoregulation is defined as the capacity of an organism to discriminate against and eliminate antigenic foreign substances, thereby maintaining homeostatic equilibrium. This process involves interactions among immune components within an integrated network, and with other systems, particularly the neuroendocrine system, to maintain systemic homeostasis. As a core physiological process, the immune response—encompassing both self-tolerance and non-self antigen rejection—is stringently governed by immunoregulatory mechanisms. These regulatory circuits are indispensable for maintaining internal milieu stability. Dysregulation of immunoregulatory functions may provoke aberrant immune attacks against self-components, leading to cellular destruction and consequent functional impairment, thereby precipitating autoimmune pathologies [30]. A suboptimal response to pathogenic microorganisms—either insufficient to clear pathogens or excessive enough to trigger hypersensitivity—may lead to severe infections or allergic reactions. Thus, the immunoregulatory mechanism governs both the initiation and magnitude of immune responses. This sophisticated regulation operates at multiple levels of the immune cascade, including antigen recognition, signal transduction, and effector cell activation [31]. Immunoregulation constitutes a sophisticated physiological process involving coordinated crosstalk among immune components and with non-immune systems to maintain homeostatic equilibrium. This mechanism orchestrates protective immunity against pathogens while imposing self-tolerance to self-tissues, thereby preserving organismal integrity [32].

Immunoregulation can be categorized into three distinct levels: self-regulation, systemic regulation, and population regulation. Self-regulation primarily pertains to interactions within the immune system, particularly those occurring between immune cells and immune molecules [33]. Systemic regulation entails interactions between the immune system and the neuroendocrine system, thereby ensuring that immune responses are initiated and maintained at optimal levels. Population regulation emphasizes the adaptive diversity of the major histocompatibility complex (MHC) within a species, as the considerable variation of MHC genes among individuals enhances the population’s overall ability to respond to a wide array of pathogens. The immune system is composed of three principal components: immune organs, immune cells, and immune molecules. Immune organs, including the tonsils, lymph nodes, thymus, spleen, and bone marrow, serve as sites for the generation, maturation, and distribution of immune cells. Immune cells primarily consist of lymphocytes and phagocytes. Lymphocytes are further classified into T cells and B cells, which mature in the thymus and bone marrow, respectively [34]. Immune molecules such as antibodies, lymphokines, and lysozymes play crucial roles in mediating immune responses. Immunity can be classified into humoral and cellular immunity. Humoral immunity primarily targets antigens that invade the body’s internal environment. B cells produce antibodies that bind specifically to these antigens, resulting in the formation of antigen–antibody complexes or agglutinated cell clumps, which are subsequently phagocytosed and degraded by immune cells [35]. Cellular immunity primarily targets host cells that have been invaded by antigens, referred to as target cells. Effector T cells interact directly with these target cells, inducing their lysis and death, and thereby facilitating the elimination of pathogens. The physiological mechanisms underlying immunoregulation involve numerous complex interactions among immune cells, molecules, and signaling pathways [36]. For example, antibodies specifically bind to antigens, thereby neutralizing pathogens and preventing them from causing further harm to the body. T cells play a central role in cellular immunity by recognizing and destroying virus-infected cells or tumor cells, thereby safeguarding the body against disease. Additionally, memory cells are essential for immunoregulation, as they enable the immune system to mount faster and more robust responses upon re-exposure to the same pathogen [37].

Nonspecific immunity refers to the body’s innate defense mechanisms, including physical barriers such as the skin and mucous membranes, as well as bactericidal substances present in body fluids. These mechanisms provide broad protection against a wide range of pathogens [38]. In contrast, specific immunity is acquired and generates immune responses that target particular pathogens, as seen in immunity developed through vaccination. Specific immunity encompasses both humoral and cellular components, which work synergistically to protect the body from pathogen invasion. When immunoregulatory functions are impaired, this can result in a range of diseases [39]. For example, immunodeficiency diseases arise from inadequate immune system function, rendering the body unable to effectively defend against pathogenic infections. Autoimmune diseases develop when the immune system mistakenly attacks the body’s own tissues, resulting in cellular damage and loss of function. Allergic reactions occur when the immune system overreacts to otherwise harmless substances, leading to tissue damage and adverse symptoms [40].

In summary, immunoregulation is a critical mechanism for maintaining human health, as it ensures that the immune system operates at an optimal level through intricate interactions and precise regulatory processes.

#### 2.3.2. Direct Immune Effects

The immunomodulatory effects of *Ganoderma lucidum* are among its most prominent pharmacological properties, encompassing the regulation of diverse immune cell functions and the activation of multiple signaling pathways. *Ganoderma lucidum* can modulate immune responses through the following mechanisms: 

(1) Enhance the function of dendritic cells (DCs), which are the most potent antigen-presenting cells in the body, responsible for capturing, processing, and presenting antigens to initiate immune responses. *Ganoderma lucidum* polysaccharides can promote the maturation, differentiation, and expression of signaling molecules in DCs, thereby enhancing their antigen uptake capacity, effectively activating naïve T cells, and strengthening immune responses. Recent scientific studies have further demonstrated that *Ganoderma lucidum* polysaccharides significantly enhance DC function by activating toll-like receptor (TLR) and NF-κB signaling pathways [27]. 

(2) Enhance the function of the mononuclear phagocyte system and natural killer (NK) cells. The mononuclear phagocyte system serves as the body’s first line of innate defense and is primarily responsible for nonspecific immune functions [41]; NK cells are the primary effectors in the immune system responsible for targeting tumor cells. *Ganoderma lucidum* has been shown to enhance the functions of both NK cells and cells of the mononuclear phagocyte system, increasing their phagocytic capacity and cytotoxic activity, thereby strengthening the body’s resistance to bacteria, viruses, and tumor cells [42]. Recent studies have shown that triterpenoids found in *Ganoderma lucidum* can activate protein kinase C (PKC) and MAPK signaling pathways, thus enhancing the activity of both monocytes and NK cells [43]. 

(3) Enhance humoral and cellular immune functions. *Ganoderma lucidum* can stimulate plasma cells, which are differentiated from B lymphocytes, to produce antibodies and thereby enhance humoral immune responses. Additionally, *Ganoderma lucidum* can increase the activity of T helper cells and cytotoxic T cells, thus improving cellular immune responses [44]. Both humoral and cellular immune responses play critical roles in anti-infection, antitumor immunity, and the elimination of foreign substances. Scientific research has demonstrated that *Ganoderma lucidum* polysaccharides can promote the secretion of interleukin-2 (IL-2) and interferon-gamma (IFN-γ) [45], thereby further enhancing both humoral and cellular immune functions [46]. 

(4) Promote the production of immune cytokines. Immune cytokines are small peptide molecules synthesized and secreted by various immune cells within the body, including interleukins (ILs), interferons (IFNs), and tumor necrosis factors (TNFs) [47] (Table 1). *Ganoderma lucidum* modulates the synthesis and secretion of these cytokines, thereby regulating immune function and enhancing the body’s resistance to diseases.

Recent studies have shown that the active components in *Ganoderma lucidum* can activate the STAT signaling pathway in immune cells, thereby promoting cytokine production [54]. Various active components in *Ganoderma lucidum*, such as polysaccharides and triterpenoids, synergistically modulate the immune system through multiple mechanisms, thereby enhancing the body’s immune defense capabilities. The immunomodulatory effects of *Ganoderma lucidum* polysaccharides are mainly exerted by binding to receptors on the surfaces of immune cells, thereby activating multiple signaling pathways [48, 49,50]. These mechanisms include the following:

(1) Activation of TLRs [55]: *Ganoderma lucidum* polysaccharides can bind to TLRs, thereby activating macrophages and dendritic cells. This activation enhances the phagocytic capacity of these cells and promotes the secretion of pro-inflammatory cytokines, ultimately strengthening the body’s immune defense. 

(2) Activation of C-type lectin-like receptors (CLRs): CLRs are important targets for polysaccharides, and *Ganoderma lucidum* polysaccharides can enhance the antigen-presenting ability of dendritic cells through these receptors, thereby promoting T cell activation and proliferation [56]. 

(3) Enhancement of specific immune responses: *Ganoderma lucidum* polysaccharides can promote the proliferation and differentiation of both T cells and B cells, thereby enhancing the capacity for specific immune responses and enabling the body to respond to pathogens more rapidly and effectively [57]. 

Compared with *Ganoderma lucidum* polysaccharides, *Ganoderma lucidum* triterpenoids primarily exert their immunomodulatory effects by inhibiting inflammatory responses and regulating immune homeostasis. The mechanisms underlying these effects include the following [58]: 

(1) Inhibition of inflammatory factors: *Ganoderma lucidum* triterpenoids can inhibit the production and release of inflammatory mediators, thereby alleviating chronic inflammatory responses in the body. Studies have shown that *Ganoderma lucidum* triterpenoids exert their anti-inflammatory effects mainly by suppressing the NF-κB and MAPK signaling pathways, which leads to a reduction in the production of inflammatory mediators.

(2) Induction of immune cell apoptosis: *Ganoderma lucidum* triterpenoids can induce apoptosis in specific immune cells, which prevents excessive activation of the immune system and helps maintain immune homeostasis. Through this mechanism, *Ganoderma lucidum* triterpenoids demonstrate therapeutic potential in the management of chronic inflammation and immune-related diseases [59,60,61].

In summary, *Ganoderma lucidum* exerts direct and comprehensive immunomodulatory effects by targeting both innate and adaptive immune cells. Its polysaccharides primarily function as immunostimulants by activating pattern recognition receptors such as TLRs and CLRs, thereby enhancing antigen presentation and promoting lymphocyte proliferation, while its triterpenoids mitigate excessive inflammation and maintain immune homeostasis through the modulation of signaling pathways such as NF-κB and MAPK. This multi-targeted and synergistic interplay between these components underlies the potent bidirectional immunomodulatory capacity of *Ganoderma lucidum*.

#### 2.3.3. Regulating Gut Microbiota

In addition to its direct effects on immune cells, *Ganoderma lucidum* can also modulate the immune system indirectly by influencing the gut microbiota. The relationship between the gut microbiota and immune regulation is especially close. The gut microbiota refers to the entire community of microorganisms residing in the intestine, and its composition and abundance are influenced by a variety of factors, including diet, environment, and genetics [62]. The gut microbiota interacts with the host immune system through its metabolic products, cell-wall components, and immune-regulatory molecules, thereby influencing the initiation and development of immune responses. For example, short-chain fatty acids produced by the gut microbiota not only provide energy for intestinal epithelial cells but also promote the generation of regulatory T cells, thereby maintaining gut immune homeostasis. Additionally, the gut microbiota can transmit signals from the gut to the central nervous system by activating the vagus nerve or producing neurotransmitters, thus affecting immune cell function [63]. The primary bioactive components of *Ganoderma lucidum* include polysaccharides, triterpenoids, oligosaccharides, and trace elements. Among these, *Ganoderma lucidum* polysaccharides and triterpenoids are the most extensively studied. *Ganoderma lucidum* polysaccharides are mainly located in the fungal cell wall and possess a complex helical structure. Their pharmacological activity is closely associated with their molecular weight [64]. *Ganoderma lucidum* triterpenoids are lipid-soluble compounds characterized by a highly oxidized lanostane derivative structure. Studies have demonstrated that *Ganoderma lucidum* polysaccharides can promote the growth of beneficial bacteria such as bifidobacteria and lactobacilli while inhibiting the proliferation of harmful bacteria, thereby enhancing the diversity and stability of the gut microbiota [34]. This enhancement of the gut microbiota not only positively regulates the host immune system but also influences brain function through the “gut—immune–brain” axis, thereby modulating mood and behavior. *Ganoderma lucidum* can also regulate the gut microbiota by promoting the growth of beneficial bacteria and inhibiting the proliferation of harmful bacteria, thereby further strengthening the gut barrier to prevent harmful substances from entering the bloodstream and impacting liver health. Specifically, the modulatory effects of *Ganoderma lucidum* on the gut microbiota are mainly reflected in the following aspects [43,55]: 

(1) Promoting the growth of beneficial bacteria: The polysaccharide components of *Ganoderma lucidum* can serve as prebiotics, supplying nutrients to beneficial bacteria such as bifidobacteria and lactobacilli in the gut, thereby stimulating their growth and proliferation. These beneficial bacteria enhance gut barrier function, suppress the growth of harmful bacteria, and help maintain the gut microbial ecological balance [65,66].

(2) Inhibiting the proliferation of harmful bacteria: The triterpenoids present in *Ganoderma lucidum* possess notable antibacterial properties and are capable of suppressing the proliferation of harmful bacteria such as Escherichia coli and Salmonella in the gut, thereby reducing the production of toxic metabolites and safeguarding gut health [48]. 

(3) Enhancing gut barrier function: *Ganoderma lucidum* polysaccharides can stimulate mucus secretion by intestinal epithelial cells, thereby reinforcing the integrity of the intestinal mucosal barrier, preventing the translocation of harmful substances into the bloodstream, alleviating the burden on the liver, and supporting the health of the gut–liver axis [67]. 

(4) Improving gut immune function: *Ganoderma lucidum* can enhance the activity of intestinal immune cells, such as macrophages and natural killer cells, thereby strengthening gut immune defenses, preventing pathogen invasion, and supporting overall gut health [62].

In summary, *Ganoderma lucidum* modulates the gut microbiota, primarily through the prebiotic effects of its polysaccharides and the antimicrobial properties of its triterpenoids. This regulation enhances microbial diversity, strengthens the intestinal barrier, and bolsters local immunity. Consequently, these gut-centric actions underpin its systemic immunomodulatory role via the gut–liver and gut–brain axes.

## 3. Mechanisms of Immunomodulation in Brain–Gut–Liver Axis Disorders and Advances in Their Treatment and Prevention

### 3.1. Mechanisms of Immunomodulation in Hepato-Intestinal Axis-Like Diseases

**Figure 2:**
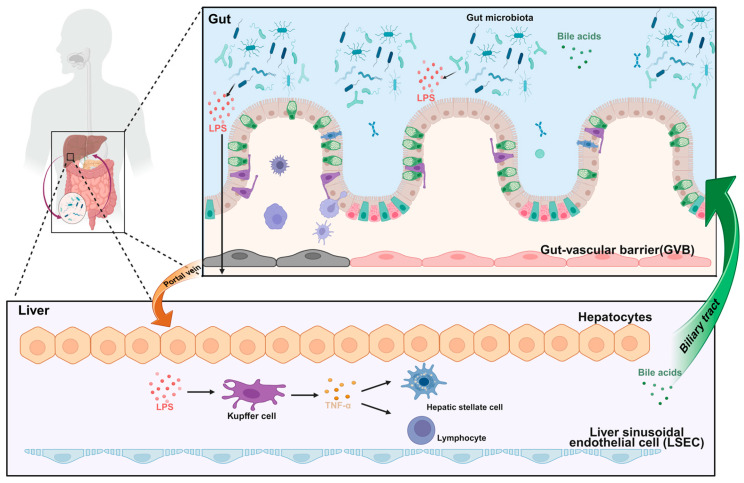
Gut–liver axis: the mechanisms of interaction between the gut microbiota and the liver. This diagram illustrates the interaction mechanisms of the gut–liver axis. The upper part depicts the gut, where the gut microbiota produce substances such as lipopolysaccharide (LPS), and the gut–vascular barrier (GVB) regulates the transport of these substances. The lower part depicts the liver, where LPS and similar compounds enter through the portal vein and act on Kupffer cells, prompting them to release tumor necrosis factor-alpha (TNF-α). This, in turn, affects hepatic stellate cells, lymphocytes, and liver sinusoidal endothelial cells (LSECs). Meanwhile, bile acids are transported via the biliary tract, participating in the mutual regulation between the gut and the liver.

The gut–liver axis constitutes a vital physiological connection between the liver and the gastrointestinal tract within the human body’s complex systemic network. This axis is integral to multiple biological functions, including digestion, absorption, substance metabolism, and detoxification, as well as immune regulation (Figure 2). Through intricate bidirectional signaling mechanisms, the gut–liver axis not only mediates metabolic and detoxification processes but also plays an indispensable role in maintaining immune homeostasis, thereby exerting profound effects on both physiological and pathological conditions [68]. By gaining a deeper understanding of the mechanisms underlying immune regulation within the gut–liver axis, we can not only enhance our comprehension of human health but also identify novel therapeutic strategies for a broad spectrum of diseases. Dysregulation of the gut–liver axis has been linked to the pathogenesis of various liver disorders, including non-alcoholic fatty liver disease (NAFLD) and cirrhosis, highlighting the clinical significance of this complex physiological system [69]. The pathogenesis of NAFLD is multifactorial and complex, with aberrant activation of the immune system representing a critical component. Impairment of the intestinal barrier permits the translocation of bacteria and their metabolites into the liver, where they elicit an exaggerated immune response. This, in turn, leads to hepatocellular injury and inflammation. The ensuing immune-mediated inflammatory response is recognized as a pivotal factor driving the progression of NAFLD to non-alcoholic steatohepatitis (NASH) [70]. Cirrhosis represents the end stage of chronic liver disease, in which dysregulated activation of the immune system plays a pivotal role in its pathogenesis.

The essence of the gut–liver axis resides in the intricate interplay between the gut microbiota and the liver. Dysbiosis of the gut microbiota, coupled with compromised intestinal barrier integrity, facilitates the translocation of bacterial endotoxins into the liver, thereby triggering immune responses that contribute to hepatic fibrosis and sclerosis [71]. As the largest microbial community in the human body, the gut microbiota exerts a profound influence on host health. It modulates numerous physiological processes through the production of diverse metabolites, including short-chain fatty acids and bile acids [72]. Among these metabolites, bile acids play a pivotal role in immune regulation within the gut–liver axis. Bile acids are synthesized by the liver and secreted into the intestine, where they primarily facilitate the digestion and absorption of lipids. Beyond their digestive functions, bile acids also serve as important signaling molecules capable of modulating immune responses [73]. Bile acids regulate inflammatory responses and immune cell activity primarily through the activation of specific receptors, such as the farnesoid X receptor (FXR) and the G protein-coupled bile acid receptor 1 (TGR5). For instance, FXR activation suppresses the production of pro-inflammatory cytokines, thereby mitigating inflammation in both the liver and the intestine. The gut microbiota plays a crucial role in bile acid metabolism, further influencing the composition and signaling functions of the bile acid pool [74]. Gut bacteria are capable of converting primary bile acids into secondary bile acids, a transformation that not only increases the diversity of the bile acid pool but also enhances their capacity for immune regulation. Secondary bile acids can further modulate host immune responses by binding to receptors such as FXR and TGR5, thereby influencing various immunological and metabolic pathways within the gut–liver axis [75]. Additionally, the gut microbiota can influence the immune status of both the liver and intestine by regulating the synthesis and reabsorption of bile acids. The immune regulatory function of the gut–liver axis is also exemplified by its role in maintaining intestinal barrier integrity. As the first line of defense against potentially harmful substances, the intestinal barrier relies on bile acids, which play a critical role in preserving its structure and function. Through their interactions with gut epithelial cells and immune cells, bile acids contribute to the maintenance of barrier homeostasis and the prevention of inflammation-related disorders [75]. Bile acids enhance the tight junctions of intestinal epithelial cells, thereby improving the integrity of the intestinal barrier and preventing the invasion of pathogens and other harmful substances [76]. In addition to their barrier-protective functions, bile acids can modulate intestinal immune responses by influencing the activity of immune cells such as T cells and B cells within the gut. The immune regulatory mechanisms of the gut–liver axis are increasingly recognized as pivotal in the pathogenesis and progression of various diseases. For example, in NAFLD, disruptions in gut microbiota composition and aberrant bile acid metabolism are considered key contributors to disease development. Therapeutic strategies aimed at modulating the gut microbiota and bile acid metabolism have been shown to ameliorate NAFLD symptoms, highlighting the potential of targeting the gut–liver axis in disease management [77]. Moreover, the gut–liver axis plays a pivotal role in the pathogenesis of a range of diseases, including inflammatory bowel disease (IBD), cirrhosis, and liver cancer. With advances in research on the gut–liver axis, the significance of immune regulation within this physiological network is becoming increasingly apparent [78]. Modulation of the gut microbiota and bile acid metabolism presents novel therapeutic avenues for the management of various diseases. For instance, supplementation with probiotics and prebiotics can optimize the composition of the gut microbiota, thereby enhancing immune function in both the liver and intestine. In addition, the development of pharmacological agents targeting bile acid receptors offers promising new strategies for the treatment of hepatic and intestinal disorders [79].

**Figure 3:**
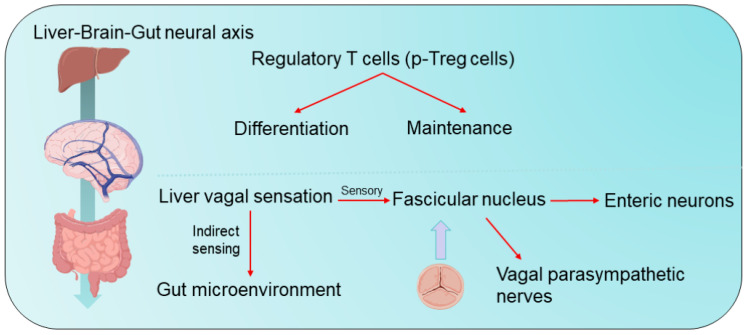
Regulatory mechanisms of the liver–brain–gut neural axis. Sensory signals from the liver’s vagus nerve are transmitted to the fascicular nucleus, which, on the one hand, acts on enteric neurons and, on the other hand, regulates the vagal parasympathetic nerves. Meanwhile, liver vagal sensation can also indirectly perceive the gut microenvironment. Additionally, regulatory T cells (p-Treg cells) participate in differentiation and maintenance processes, collectively forming the neural regulatory network between the liver, brain, and gut.

In recent years, a novel gut–liver–brain neural axis has been identified that is essential for the proper differentiation and maintenance of regulatory T cells (Treg cells) in the gut [80]. Specifically, the sensory afferent fibers of the hepatic branch of the vagus nerve are capable of indirectly sensing the gut microenvironment and relaying this sensory information to the nucleus tractus solitarius in the brainstem, which subsequently transmits signals to vagal parasympathetic nerves and enteric neurons. This newly described vagal gut–liver–brain reflex arc can regulate the number of peripheral Treg (p-Treg) cells, maintain intestinal homeostasis, and is of great importance for the prevention and treatment of immune-mediated diseases of the gut. Furthermore, postbiotics—the metabolic products of the gut microbiota—also play a significant role in immune regulation [81]. Postbiotics have been shown to promote the growth and proliferation of beneficial gut bacteria while inhibiting the expansion of harmful microbial populations, thereby improving the overall intestinal environment. In addition, postbiotics enhance the repair and regeneration of the intestinal mucosal barrier, increasing the gut’s resistance to external pathogens and harmful substances. Moreover, the administration of postbiotics can activate intestinal immune cells and boost immune function, offering both preventive and adjunctive therapeutic benefits for conditions such as intestinal inflammation, allergies, and other related diseases (Figure 3).

Additional studies have elucidated the intricate relationship between IBD and psychological stress [82]. IBD, an autoimmune gastrointestinal disorder, is characterized by chronic inflammation and recurrent relapses. Increasing evidence shows that chronic psychological stress serves as a significant trigger for the exacerbation and recurrence of IBD. Furthermore, the bidirectional nature of the gut–brain axis [83] has been emphasized, indicating that the pathophysiological processes of IBD can exacerbate inflammatory responses within the central nervous system, thereby contributing to behavioral comorbidities such as anxiety and depression. This body of evidence provides a more comprehensive understanding of the association between psychological stress and disease activity in IBD, offering valuable insights into the underlying mechanisms linking emotional states with chronic gastrointestinal inflammation.

(1) Relationship between the gut microbiome and IBD [82]: Although the precise triggers of immune responses in IBD remain unclear, even with the advent of highly effective biologic therapies in recent decades, it is noteworthy that the majority of immune cells are situated within the gut. Dysregulation of the gut microbiome, therefore, can have far-reaching effects that extend beyond the gastrointestinal tract. This observation suggests that the gut microbiome may play a crucial role in the pathogenesis of diseases involving the gut–liver axis, such as IBD. 

(2) Gut microbiome changes in psoriasis patients [83]: Studies have demonstrated that the gut microbiome composition in patients with psoriasis differs significantly from that of healthy individuals at the levels of genera, species, functional pathways, and microbial network complexity [82]. Furthermore, the Microbiome Dysbiosis Index has emerged as a potentially cost-effective and rapid tool for monitoring probiotic interventions in patients with psoriasis, providing valuable insights into microbial alterations associated with disease progression and therapeutic response. 

(3) Comparison of gut microbiomes of patients with psoriasis and healthy subjects: A comparative analysis was conducted involving 58 patients with psoriasis and 49 healthy local individuals who were presumed to share a similar lifestyle [84]. The results revealed that specific microbial taxa were differentially enriched between the two groups. In particular, certain microbial fractions were predominantly found in psoriasis patients, while others were more abundant in healthy subjects. Functional predictions indicated distinct enrichments in metabolic pathways, with healthy individuals exhibiting a marked preference for the synthesis of short-chain fatty acids (SCFAs) [85]. This suggests that alterations in gut microbiome composition and function may be associated with the pathogenesis of psoriasis. Alterations in co-occurring microbial networks were also observed in patients with psoriasis. 

In summary, recent advances in understanding the immunomodulatory roles within diseases of the gut–liver axis underscore a strong association between the gut microbiome and chronic immune-mediated inflammatory diseases, such as IBD and psoriasis [86]. Modulation of the gut microbiome may therefore offer novel strategies for the treatment and management of these conditions. Future research should focus on elucidating the complex mechanisms underlying the interactions between the gut microbiome and gut–liver axis diseases, as well as exploring how these insights can be leveraged to develop more effective therapeutic approaches.

In summary, immune dysregulation within the gut–liver axis, driven by gut barrier disruption, dysbiosis, and altered bile acid signaling, is a critical driver in the pathogenesis of NAFLD, cirrhosis, and IBD. The recently identified gut–liver–brain neural circuit further expands this axis, highlighting neural control over intestinal immune tolerance. Targeting these interconnected immune mechanisms offers promising therapeutic avenues for managing gut–liver axis disorders.

### 3.2. Mechanisms of Immunomodulation in Gut–Brain Axis Disorders

The gut–brain axis refers to the bidirectional communication network that exists between the brain and the gastrointestinal tract, which plays a pivotal role in regulating digestive functions, emotional states, and immune responses [87]. Recent research has demonstrated that dysregulation of the gut–brain axis is closely linked to the development of various disorders, including irritable bowel syndrome (IBS), depression, and autism spectrum disorder (ASD) [88]. IBS is a prevalent functional gastrointestinal disorder with an unclear etiology; however, abnormalities in the immune system are recognized as one of the key pathogenic mechanisms. Evidence shows that patients with IBS display heightened activity of immune cells within the intestinal mucosa, accompanied by excessive release of inflammatory mediators [89]. This state of immune activation may compromise the integrity of the gut barrier, thereby triggering hypersensitivity reactions and abdominal pain [90]. In patients with depression and ASD, imbalances in the gut microbiota are closely associated with abnormal activation of the immune system. Gut microbes can influence central nervous system function by producing various metabolites, thereby modulating emotions and behavior [88]. Emerging evidence shows that dysbiosis of specific gut bacterial populations may provoke aberrant immune responses, which in turn can impact brain function.

**Figure 4:**
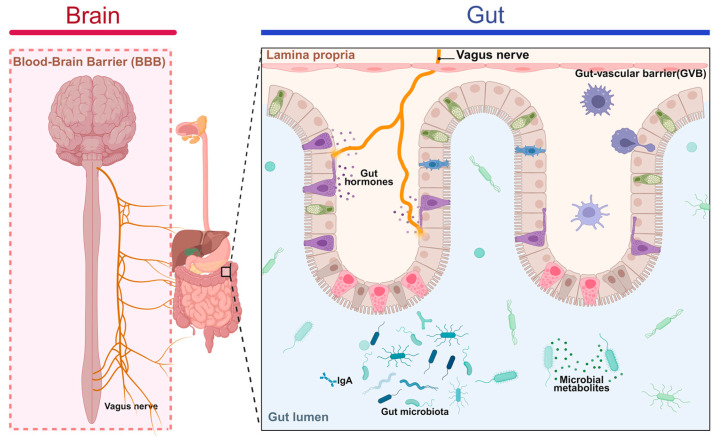
Gut–brain axis: the bidirectional communication mechanism between the brain and the gut. The left side shows the brain and the blood–brain barrier (BBB), with the vagus nerve connecting the brain to the gut. The right side presents the local structure of the gut, including intestinal villi, the lamina propria, and the gut–vascular barrier (GVB). The gut microbiota, microbial metabolites, and gut hormones interact with the brain through pathways such as the vagus nerve and humoral routes. This demonstrates the complex bidirectional transmission of information and the regulatory relationship between the brain and the gut.

The gut–brain axis is a sophisticated bidirectional communication system that links the brain and the gastrointestinal tract, regulating a wide range of physiological functions through the coordinated interactions of the nervous, endocrine, and immune systems [91]. Among these, immune regulation serves as a critical component, playing an essential role in maintaining the health of both the gut and the brain [92]. Firstly, the vagus nerve, as a principal pathway of the gut–brain axis, plays a pivotal role in immune regulation [89]. It transmits signals from the gut to the brain, targeting regions such as the cerebral cortex, hypothalamus, and nucleus of the solitary tract, thereby modulating the activity of immune cells (Figure 4). Studies have demonstrated that the vagus nerve can enhance systemic immune responses by activating macrophages in the spleen, which, in turn, promotes antibody production [93]. Additionally, vagus nerve stimulation has been shown to reduce the release of inflammatory mediators [94], thereby helping to suppress inflammatory responses. Furthermore, intestinal epithelial cells, as an essential component of the gut–brain axis, serve as a bridge between the gut microbiota and the immune system by secreting hormones, neurotransmitters, and cytokines [91]. Through these mechanisms, intestinal epithelial cells facilitate complex interactions that are crucial for maintaining immune homeostasis within both the gut and the central nervous system. Beyond serving as a physical barrier that protects the gut from harmful substances, intestinal epithelial cells also regulate immune responses through dynamic interactions with the gut microbiota [78]. Metabolites produced by the gut microbiota, such as SCFAs, can activate dendritic cells and macrophages within the lamina propria of the gut mucosa. This activation induces the production of both pro-inflammatory and anti-inflammatory cytokines, thereby modulating mucosal immune responses and contributing to the overall regulation of immune homeostasis [95]. The gut–brain axis involves intricate interactions among the nervous, immune, and endocrine systems, with immune regulation serving as a critical component [94]. Emerging evidence shows that disruptions in the gut–brain axis are associated with the pathogenesis of various diseases [96], including IBD, multiple sclerosis (MS), and ASD [97]. In these conditions, the immune regulatory mechanisms mediated by the gut–brain axis may encompass modulation of the gut microbiota, activation of neuroimmune pathways [98], and regulation of the neuroendocrine system [99]. These processes collectively contribute to disease development and progression, highlighting the significance of gut–brain axis homeostasis in maintaining overall health. 

Gut microbiota and immune regulation: The gut microbiota exerts profound effects on immune regulation and brain function through multiple mechanisms [100]. It can modulate the host’s immune response, influencing inflammatory processes within the gut, systemically, and even within the brain via neuroinflammation [94]. Furthermore, the gut microbiota may indirectly impact brain function by regulating the synthesis of neurotransmitters such as serotonin, and by activating neuroendocrine pathways, including the hypothalamic–pituitary–adrenal axis [98]. 

Neuroimmune interactions: Within the gut, both the immune system and the enteric nervous system (ENS) play vital roles in monitoring and maintaining the boundaries between symbiotic and pathogenic microbes [100]. These neuroimmune interactions are essential for preserving intestinal homeostasis and protecting against infection while also contributing to the broader communication network of the gut–brain axis. Recent research has demonstrated that regulatory T cells (Tregs) functionally interact with the enteric nervous system (ENS), thereby influencing immune tolerance and maintaining gut homeostasis [94]. Moreover, microbial-derived signals have been shown to regulate both the density and activation of enteric nerves, which, in turn, modulate the generation of Tregs and the establishment of immune tolerance. 

Application of immune regulation in neuropsychiatric disorders: Emerging evidence shows that the gut microbiota participates in the regulation of emotional behaviors, stress responses, and inflammatory processes. However, the precise neurobiological mediators underlying these effects remain to be fully elucidated [101]. For example, peroxisome proliferator-activated receptor alpha (PPAR-α) is a transcription factor that regulates various pathophysiological processes [102], including metabolic pathways and immune responses. Its modulatory effects are, in part, mediated through epigenetic modifications, which influence gene expression and cellular function [103]. 

Application of immune regulation in neurodegenerative diseases: In neurodegenerative disorders such as Alzheimer’s disease, mounting evidence shows that both the gut microbiota and the immune system contribute to disease pathogenesis and clinical manifestations [104]. Recent studies have highlighted the involvement of central nervous system (CNS)-resident and peripheral immune pathways in the bidirectional communication within the microbiota–gut–brain axis. 

Traumatic brain injury (TBI) and immune regulation [90]: Traumatic brain injury is increasingly recognized as a chronic, progressive disorder that involves not only the primary neural insult but also a range of secondary effects, including immune-mediated alterations impacting peripheral organs such as the gastrointestinal tract [85,105]. The gut–brain axis comprises bidirectional communication pathways through which TBI-induced neuroinflammation and neurodegeneration can disrupt gut function and homeostasis. 

Treatment strategies [102]: In light of these interactions, researchers are actively investigating therapeutic approaches that target immune regulation within the gut–brain axis. These strategies include interventions aimed at modulating the composition of the gut microbiota, such as the administration of probiotics, prebiotics, and dietary modifications, with the goal of mitigating TBI-associated neuropathology and improving clinical outcomes [106]. Additionally, therapeutic strategies targeting the gut–brain axis may also include interventions aimed at alleviating neuroinflammation and demyelination [101]. The ongoing research and application of immune regulation within the gut–brain axis represent a multidisciplinary and rapidly expanding field [88], spanning from basic scientific investigation to the development and clinical application of novel treatment modalities [92]. As our understanding of the complex interplay between the gut–brain axis and immune regulation continues to deepen, it is anticipated that an increasing number of targeted therapies for neurological and psychiatric disorders will emerge in the future.

In summary, immunomodulation plays a pivotal role within both the gut–brain and gut–liver axes. Through complex interactions involving the vagus nerve, intestinal epithelial cells, gut microbiota, and their metabolites, immune regulation not only maintains intestinal homeostasis but also exerts profound effects on brain function and development. In-depth exploration of the immune regulatory mechanisms underlying the gut–brain and gut–liver axes offers novel insights and approaches for understanding, preventing, and treating a wide spectrum of intestinal and neurological disorders. Advancing our knowledge in this area will not only enhance our comprehension of human health but also facilitate the development of innovative strategies and therapeutic interventions. Looking forward, continued research on the gut–brain and gut–liver axes is expected to yield new opportunities and breakthroughs that will significantly benefit human health.

### 3.3. Treatment and Prevention of Gut–Brain Axis and Gut–Liver Axis Disorders

Currently, research on gut–brain axis disorders predominantly focuses on modulating the balance between the gut microbiota and the immune system. The administration of probiotics and prebiotics is recognized as an effective strategy to regulate gut microbial composition and has been shown to alleviate symptoms in patients with IBS [107]. In addition, psychotherapy and antidepressant medications have demonstrated certain efficacy in the treatment of depression and ASD. In the context of gut–liver axis disorders, recent advances have centered on enhancing gut barrier function and modulating immune responses [108]. The use of probiotics, prebiotics, and dietary fibers is believed to strengthen gut barrier integrity and decrease the translocation of bacterial endotoxins [109]. Moreover, antioxidants and anti-inflammatory agents have shown therapeutic potential in the management of NAFLD and liver cirrhosis.

## 4. *Ganoderma lucidum*: The Immune Guardian of the Gut–Liver and Gut–Brain Axes

### 4.1. Ganoderma lucidum Prevents and Treats Diseases by Regulating the Immune System

In today’s fast-paced society, immunity has become a crucial line of defense for maintaining our health. *Ganoderma lucidum*, an ancient Chinese medicinal herb, not only exhibits remarkable efficacy in enhancing immune function [110] but also plays a vital role in the immunomodulation of both the gut–liver and gut–brain axes. The gut–liver axis is a complex physiological network in which the liver and intestines are closely interconnected via blood and lymphatic circulation, as well as neuroendocrine pathways [111]. Studies have demonstrated that *Ganoderma lucidum* polysaccharides can promote the repair and regeneration of liver cells, enhance the liver’s capacity to metabolize harmful substances, and thereby help maintain the health of the gut–liver axis [112]. The gut–brain axis is a neuroendocrine network that links the brain and the intestines, influencing mood, cognitive function, and immune responses [113]. *Ganoderma lucidum* also plays an important role in regulating the gut–brain axis. The bioactive compounds contained in *Ganoderma lucidum* exhibit potent anti-inflammatory and antioxidant properties, which help reduce neuroinflammation, enhance cerebral blood circulation, and facilitate the repair and regeneration of nerve cells [114,115]. *Ganoderma lucidum* also modulates the secretion of neurotransmitters such as serotonin (5-hydroxytryptamine) and dopamine, thereby improving mood, alleviating symptoms of anxiety and depression, and enhancing sleep quality [116]. These effects not only help maintain the balance of the gut–brain axis but also enhance the body’s resilience to stress and strengthen overall immunity.

**Figure 5:**
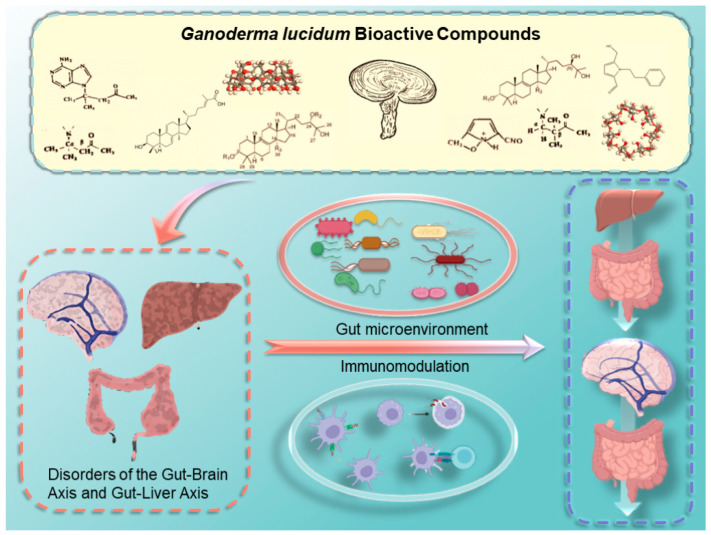
*Ganoderma lucidum* treats liver–intestinal axis and brain–intestinal axis-like diseases by regulating the immune system and intestinal flora. The top section displays a variety of bioactive compounds derived from *Ganoderma lucidum*. These components exert beneficial effects on the brain, gut, and liver functions by modulating the gut microenvironment (e.g., microbial composition) and systemic immune responses (central section). This model integrates the chemical diversity of *Ganoderma lucidum*, its regulatory effects on the gut microbiota and the immune system, and its potential to ameliorate disorders associated with the gut–brain and gut–liver axes, demonstrating its multi-target characteristics.

The immunomodulatory effects of *Ganoderma lucidum* play a crucial role in both the gut–liver and gut–brain axes. It stimulates the activity of immune cells such as macrophages and natural killer cells, thereby enhancing the body’s defense against pathogens, including viruses and bacteria [117]. *Ganoderma lucidum* also promotes the secretion of cytokines, such as interleukins and interferons, which help regulate immune responses and prevent autoimmune diseases resulting from excessive immune activation [38]. In addition, the bidirectional regulation of the immune system by *Ganoderma lucidum* allows it to help maintain immune system stability during both immunodeficiency and hyperactive immune responses [118]. Modern scientific research has identified polysaccharides and triterpenoids as the primary active components of *Ganoderma lucidum*. Polysaccharides exhibit antitumor, antiviral, and immune-enhancing properties, whereas triterpenoids demonstrate anti-inflammatory, antioxidant, and hepatoprotective effects [119]. For example, one study indicated that *Ganoderma lucidum* polysaccharides significantly improved immune function in mice, increased the weights of the spleen and thymus, and enhanced immune cell activity [120]. In addition, the regulatory effects of *Ganoderma lucidum* on the gut–brain axis have also been supported by scientific evidence [121]. A study published in the journal *Frontiers in Pharmacology* reported that triterpenoids from *Ganoderma lucidum* were effective in reducing neuroinflammation, improving cognitive function [122], and combating symptoms of depression and anxiety [123]. These findings provide a scientific basis for the application of *Ganoderma lucidum* in regulating the gut–brain axis (Figure 5).

The gut–brain axis refers to the bidirectional communication pathway between the brain and the gut, playing a crucial role in regulating mood, behavior, and digestive health. Through its immunomodulatory effects, *Ganoderma lucidum* may actively contribute to the improvement of disorders associated with the gut–brain axis [124]. The polysaccharides and triterpenoids of *Ganoderma lucidum* may positively influence mood and cognitive function by modulating the gut microbiota and the immune system. Studies have demonstrated that *Ganoderma lucidum* improves the intestinal environment by increasing the abundance of beneficial bacteria and inhibiting the growth of harmful bacteria. These changes in the gut microbiota not only directly enhance intestinal health but also indirectly improve brain function via the gut–brain axis [10]. IBS is a common functional gastrointestinal disorder that is frequently associated with intestinal microbiota dysbiosis and immune system dysfunction. *Ganoderma lucidum* may help alleviate the symptoms of IBS through its anti-inflammatory and immunomodulatory properties. The polysaccharides of *Ganoderma lucidum* can enhance intestinal barrier integrity and decrease the release of inflammatory mediators, thereby alleviating intestinal hypersensitivity and pain. The gut–liver axis refers to the network of interactions between the liver and the gut, which plays a vital role in metabolism, detoxification, and immunoregulation. *Ganoderma lucidum* has also demonstrated significant potential in modulating gut–liver axis disorders. Furthermore, *Ganoderma lucidum* may contribute to the prevention and amelioration of NAFLD by regulating the gut microbiota and enhancing immune function [51]. The polysaccharides of *Ganoderma lucidum* can improve intestinal barrier integrity, thereby reducing the translocation of intestinal bacteria and their metabolites into the liver and decreasing hepatic inflammatory responses. In addition, the antioxidant properties of *Ganoderma lucidum* help reduce oxidative damage to hepatocytes and prevent the progression of NAFLD [125]. In the management of cirrhosis, the immunomodulatory effects of *Ganoderma lucidum* may contribute to slowing disease progression. The triterpenoids of *Ganoderma lucidum* may attenuate hepatic fibrosis and cirrhosis by inhibiting inflammatory mediators and mitigating excessive immune responses. Meanwhile, the polysaccharides of *Ganoderma lucidum* improve intestinal health by modulating the gut microbiota and reducing liver injury caused by bacterial endotoxins. Studies have demonstrated that the polysaccharides and triterpenoids of *Ganoderma lucidum* are its primary bioactive components, exhibiting diverse functions, including immunomodulatory, antitumor, and antiviral activities [52,126]. *Ganoderma lucidum* polysaccharides exert their immunomodulatory effects by enhancing the activity of antigen-presenting cells, the mononuclear phagocyte system, and both humoral and cellular immune responses [52]. Specifically, the β-glucan present in *Ganoderma lucidum* can bind to Dectin-1 receptors on the surfaces of immune cells, activating downstream signaling pathways and consequently inducing cytokine production and enhancing immune responses [52]. For example, the binding of β-glucan initiates the activation of MAPK and NF-κB signaling pathways, both of which play pivotal roles in cytokine production and immune regulation [52]. In addition, *Ganoderma lucidum* has been shown to enhance the cytotoxic activity of NK cells and promote the secretion of perforin and granzyme, thereby strengthening immune surveillance against tumor cells [127]. In a mouse model, administration of *Ganoderma lucidum* extract significantly enhanced NK cell activity, suggesting its potential utility in antitumor immunity [126,128]. The immunomodulatory properties of *Ganoderma lucidum* are also evident in its impact on macrophages. Polysaccharides derived from *Ganoderma lucidum* have been shown to stimulate RAW264.7 mouse macrophages to secrete various cytokines, including tumor necrosis factor-α (TNF-α) and interleukin-6 (IL-6), both of which play crucial roles in inflammatory and immune responses [127]. Moreover, *Ganoderma lucidum* extract can attenuate the inflammatory response and enhance its immunomodulatory effects through the inhibition of the NF-κB and MAPK signaling pathways [128,129].

In conclusion, *Ganoderma lucidum* modulates the immune system through multiple mechanisms, such as enhancing NK cell activity, promoting the secretion of macrophage-derived factors, and regulating relevant signaling pathways. These properties highlight its considerable potential for applications in both traditional medicine and modern drug development.

### 4.2. Ganoderma lucidum Prevents and Treats Diseases by Regulating Intestinal Flora

The influence of *Ganoderma lucidum* on the intestinal microbiota within the brain–gut and gut–liver axes is primarily manifested in the following aspects [120]: 

(1) Regulation of intestinal microbiota balance: Polysaccharides and triterpenoids in *Ganoderma lucidum* help modulate the composition and balance of the intestinal microbiota [130]. Studies have demonstrated that *Ganoderma lucidum* can stimulate the growth of beneficial bacteria such as Bifidobacterium and Lactobacillus while inhibiting the proliferation of pathogenic bacteria. This rebalancing of the intestinal microbiota contributes to improved intestinal health and enhanced mucosal immunity [131]. For example, one study reported that *Ganoderma lucidum* extract significantly increased the abundance of Bifidobacterium in a mouse model and reduced the population of potentially pathogenic bacteria, thereby improving the overall composition of the intestinal microbiota. 

(2) Maintenance of intestinal mucosal integrity and enhancement of immune function: *Ganoderma lucidum* polysaccharides help maintain the structural integrity of intestinal mucosal tissues and enhance mucosal immune function, thereby protecting the intestinal tract against harmful substances. This protective effect is crucial for the prevention of intestinal diseases. According to one study [132], *Ganoderma lucidum* polysaccharides have been shown to significantly upregulate the expression of tight junction proteins in intestinal mucosal cells, thereby strengthening the intestinal barrier function. 

(3) Reduction of intestinal inflammatory response: The bioactive components of *Ganoderma lucidum* possess anti-inflammatory properties that can attenuate intestinal inflammation and help prevent dysbiosis of the intestinal microbiota [133]. Studies have demonstrated that aqueous extract of *Ganoderma lucidum* (WEGL) can modulate the composition of the intestinal microbiota and reduce inflammatory markers in high-fat diet-induced obese mice [134]. For example, one study found that WEGL-treated mice had significantly lower levels of intestinal inflammatory markers and improved intestinal flora diversity [135]. 

(4) Improvement of metabolic disorders: *Ganoderma lucidum* exerts ameliorative effects on metabolic disorders such as obesity and diabetes mellitus by modulating the intestinal microbiota [125]. Studies have found that *Ganoderma lucidum* can increase the abundance of beneficial bacteria and suppress the proliferation of harmful bacteria in the intestinal tract, thereby enhancing the body’s metabolic function [136]. A clinical study demonstrated that obese patients receiving *Ganoderma lucidum* extract exhibited significant reductions in body weight and blood glucose levels within 12 weeks [137]. 

(5) Improvement in immunity [138]: Polysaccharides and triterpenoids in *Ganoderma lucidum* exhibit significant immunomodulatory effects, which can enhance immune function and improve the body’s resistance to diseases. The intestinal tract is the largest immune organ in the human body, and the balance of the intestinal microbiota is essential for maintaining normal immune function [139]. Studies have shown that *Ganoderma lucidum* polysaccharides can activate macrophages and enhance the activity of NK cells, thereby boosting overall immune function [140].

Despite the numerous benefits of *Ganoderma lucidum* for the intestinal microbiota, individual responses to *Ganoderma lucidum* may vary [130]. Some individuals may experience gastrointestinal symptoms such as abdominal pain and diarrhea after consuming *Ganoderma lucidum*. This is because certain components in *Ganoderma lucidum* can stimulate the intestinal tract and increase bowel movements, potentially leading to gastrointestinal discomfort. In conclusion, *Ganoderma lucidum*, a valuable Chinese medicinal herb rich in various bioactive compounds, exerts a significant regulatory effect on the intestinal microbiota. By regulating the intestinal microbiota, *Ganoderma lucidum* can improve intestinal health, enhance immune function, reduce inflammatory responses, and ameliorate metabolic disorders. Studies have demonstrated that *Ganoderma lucidum* polysaccharides can modulate the composition of the intestinal microbiota and promote microecological balance [141]. In the intestinal tract, *Ganoderma lucidum* polysaccharides can promote the proliferation of beneficial microorganisms, such as bifidobacteria and lactobacilli, and inhibit the growth of harmful bacteria, including *Escherichia coli* and *Salmonella* [34]. This modulatory effect helps restore the normal balance of intestinal microbiota and reduces inflammatory responses, thereby alleviating the symptoms of enteritis. Additionally, *Ganoderma lucidum* polysaccharides can further mitigate enteritis by modulating immune system activity [142]. The occurrence of enteritis is often associated with immune system dysregulation, particularly an imbalance between Th17 cells and Treg cells. *Ganoderma lucidum* polysaccharides can inhibit the overactivation of Th17 cells while promoting the differentiation and function of Treg cells, thereby regulating immune responses and reducing intestinal inflammation [143]. Studies have shown that *Ganoderma lucidum* polysaccharides can significantly reduce the levels of inflammation and promote the repair of intestinal mucosal damage in animal models of ulcerative colitis [144]. The triterpenoids in *Ganoderma lucidum* also exhibit significant anti-inflammatory effects. They can inhibit the production of several inflammatory mediators, including tumor necrosis factor-α (TNF-α), interleukin-6 (IL-6), and interleukin-1β (IL-1β), thereby attenuating the inflammatory response [133,145]. In addition, triterpenoids inhibit the activation of the NF-κB signaling pathway, thereby further suppressing the production and release of inflammatory mediators [146,147]. *Ganoderma lucidum* is also capable of enhancing overall immunity. Its polysaccharides and triterpenoids can activate immune cells such as macrophages and natural killer cells [143], enhancing their phagocytic and cytotoxic activities and thereby increasing the body’s capacity to resist diseases [148]. This is particularly important for patients with enteritis, as enhanced immunity helps reduce intestinal infections and prevent recurrence of enteritis.

In conclusion, *Ganoderma lucidum* exerts multifaceted immunomodulatory effects on enteritis through its rich bioactive components. Its polysaccharides can regulate the intestinal flora and improve the intestinal microecological balance while also reducing intestinal inflammation by modulating the immune system. The triterpenoids in *Ganoderma lucidum* possess potent anti-inflammatory properties, inhibiting the production and release of inflammatory mediators. In addition, *Ganoderma lucidum* exhibits antioxidant effects, which help reduce oxidative stress-induced damage to the intestinal mucosa and enhance the body’s immune function. These effects make *Ganoderma lucidum* a promising adjunctive therapy for the prevention and treatment of enteritis.

## 5. Prospects and Challenges

*Ganoderma lucidum* exerts immunomodulatory effects on both the gut–liver axis and the gut–brain axis, not only by directly enhancing immune function but also indirectly through the improvement of hepatic and intestinal health. This comprehensive regulatory capacity confers *Ganoderma lucidum* with unique advantages in supporting human health. In the context of various health challenges posed by modern lifestyles, *Ganoderma lucidum* serves as a natural guardian, providing effective immune support. Whether by promoting liver and intestinal function or by regulating the balance between the brain and the gut, *Ganoderma lucidum* plays a significant role in maintaining health and preventing disease. Gut–brain axis disorders (which involve interactions between the nervous and digestive systems), such as IBS, depression, and anxiety, may be ameliorated by *Ganoderma lucidum*’s capacity to modulate immune responses and regulate the composition of the intestinal microbiota. Similarly, gut–liver axis disorders, including NAFLD, liver fibrosis, and cirrhosis, may benefit from *Ganoderma lucidum*’s ability to improve the gut microbiota, enhance immune function, and exert antioxidant effects. Thus, *Ganoderma lucidum* holds promise as a therapeutic agent for the prevention and management of a range of disorders related to the gut–brain and gut–liver axes.

### 5.1. Potential to Alleviate Mood and Improve Mental Health

*Ganoderma lucidum* polysaccharides can improve both physiological and psychological health by modulating the composition of the intestinal microbiota and increasing the proportion of beneficial bacteria, thereby exerting positive effects on the brain through the gut–brain axis. Future research may further explore the potential of *Ganoderma lucidum* for patients with depression and anxiety disorders. Notably, its anti-inflammatory and antioxidant properties may help attenuate stress-induced neuroinflammation, which could contribute to the amelioration of mood disorders. These findings suggest that *Ganoderma lucidum* holds promise as a complementary therapeutic agent for improving mental health by targeting the gut–brain axis.

### 5.2. Prospects for Application in Irritable Bowel Syndrome (IBS) and Other Functional Gastrointestinal Disorders

The anti-inflammatory and immunomodulatory properties of *Ganoderma lucidum* confer significant potential for its application in chronic gastrointestinal disorders, such as IBS. Future studies should focus on determining the effective dosage and optimal administration protocols of *Ganoderma lucidum* in IBS patients, as well as evaluating its feasibility as an adjunctive therapy for functional gastrointestinal disorders. Additionally, *Ganoderma lucidum*’s ability to enhance intestinal barrier function and reduce pro-inflammatory mediators may contribute to the alleviation of IBS-related symptoms. These findings suggest that *Ganoderma lucidum* could serve as a promising therapeutic agent in the management of chronic gastrointestinal diseases.

### 5.3. Potential for Prevention and Management of Non-Alcoholic Fatty Liver Disease (NAFLD)

*Ganoderma lucidum* can prevent the translocation of bacterial metabolites into the bloodstream and subsequent hepatic inflammatory responses by modulating intestinal barrier function, thereby demonstrating considerable potential in the prevention and management of NAFLD [125]. Future research could focus on elucidating the effects of *Ganoderma lucidum* on liver function parameters in NAFLD patients, such as liver enzyme activity and lipid metabolism, to clarify its specific therapeutic benefits. Furthermore, the antioxidant properties of *Ganoderma lucidum* may help mitigate oxidative stress within hepatocytes, thereby inhibiting the progression of liver fibrosis. Collectively, these mechanisms underscore the promise of *Ganoderma lucidum* as an adjunctive intervention in the management of NAFLD.

### 5.4. Potential to Alleviate Liver Fibrosis and Cirrhosis

In the management of liver fibrosis and cirrhosis, the anti-inflammatory and immunomodulatory properties of *Ganoderma lucidum* may contribute to reducing hepatic fibrosis and slowing disease progression. Future research should further investigate the therapeutic potential of *Ganoderma lucidum* in patients with chronic liver disease, particularly its specific effects on decreasing fibrosis biomarkers and mitigating hepatic injury. Additionally, studies evaluating the combined use of *Ganoderma lucidum* with established antifibrotic agents are warranted to advance its clinical application in the prevention and treatment of liver cirrhosis. Collectively, these efforts may help to establish *Ganoderma lucidum* as a valuable adjunct in the therapeutic arsenal against chronic liver diseases.

### 5.5. Multifunctional Mechanism of Action of Ganoderma lucidum in Combination with Precision Medicine

The multifaceted mechanisms of action exhibited by *Ganoderma lucidum*—including immunomodulation, antioxidation, anti-inflammation, and regulation of the intestinal microbiota—form a strong theoretical basis for its future application in precision medicine. By integrating data regarding a patient’s genetic background, gut microbiome, and metabolic profile, *Ganoderma lucidum* holds promise as a key component in personalized therapeutic strategies: 

(1) Personalized therapy guided by microbiome analysis: *Ganoderma lucidum* significantly influences gut microbiota composition, and its individualized application, informed by detailed microbiome profiling, offers a promising approach for future clinical practice. For example, precise analysis of a patient’s intestinal flora could guide the selection of optimal dosing and treatment regimens for *Ganoderma lucidum*, thereby delivering more tailored and effective outcomes—particularly for diseases closely associated with gut dysbiosis, such as IBS and NAFLD. 

(2) Precision therapy based on molecular biomarkers: In future clinical applications, the efficacy of *Ganoderma lucidum* may be accurately monitored via the measurement of molecular biomarkers, including inflammatory cytokines, oxidative stress markers, and immune cell activity. This biomarker-guided approach will enhance the personalization of *Ganoderma lucidum* usage across various diseases, maximizing therapeutic efficacy while minimizing adverse effects. 

(3) In-depth research into molecular mechanisms and formulations: Further studies are warranted to elucidate the specific molecular mechanisms through which *Ganoderma lucidum* modulates the gut–brain axis and gut–liver axis, with particular emphasis on its interactions with the immune system and gut microbiota. Moreover, a systematic comparison and clarification of the immunopharmacological mechanisms of various minor constituents in *Ganoderma lucidum* (such as nucleotides and sterols) represent a critical area for future research, which is essential for comprehensively elucidating its efficacy and advancing its precision medicine applications. 

Collectively, these strategies highlight the potential of *Ganoderma lucidum* as an integral tool in the advancement of precision medicine, facilitating more precise, effective, and individualized management of complex diseases linked to immune and metabolic dysregulation.

## Figures and Tables

**Table 1:** Immunomodulatory effects and mechanisms of *Ganoderma lucidum* active components.

Active Component	Effect	Mechanism	Target	References
Polysaccharides	Activate immune cells (macrophages, DCs, NK cells)	Combined with TLR receptors, they promote cytokine secretion and enhance phagocytic function.	TLR4, Dectin-1, NF-κB, MAPK	[24,25,48,49,50,51]
	Enhance humoral immunity and cellular immunity	Promote IL-2 and IFN-γ secretion; stimulate B/T cell proliferation and differentiation	IL-2/IFN-γ signaling axis	[45,46]
Triterpenoids	Anti-inflammatory, antioxidant	Inhibit NF-κB/MAPK pathways, reducing the release of inflammatory factors	NF-κBPKC, MAPK	[26,52]
	Induce immune homeostasis	Regulate Treg cell activity and inhibit excessive Th17 activation	Achieving a healthy balance between Treg and Th17 cells	[53]

Note: DC = dendritic cells; TLR = toll-like receptor; Treg = regulatory T cells.

## Data Availability

No new data were created or analyzed in this study. Data sharing is not applicable to this article.
